# Regarding ‘childhood respiratory risk profiles associate with lung function and COPD among the old population’

**DOI:** 10.1080/07853890.2025.2482864

**Published:** 2025-03-20

**Authors:** Xiaoyan Hu, Peng Sun

**Affiliations:** Center for Rehabilitation Medicine, Rehabilitation & Sports Medicine Research Institute of Zhejiang Province, Department of Rehabilitation Medicine, Zhejiang Provincial People’s Hospital (Affiliated People’s Hospital), Hangzhou Medical College, Hangzhou, Zhejiang, China

To the editor

We read the article with great interest by Qin et al. [1], which studied childhood respiratory risk profiles associated with lung function and COPD among the old population. This study concluded that people exposed to dyspnea early in life experienced greater lung function decline and increased risk of COPD later in life. We believed that there were several limitations to this study.

The assessment of respiratory risk in childhood relied on self-recall (e.g. did you have asthma before age 16) among older participants, which may have led to inaccurate data (e.g. forgetfulness or misreporting). Sample sizes for some risk profile groups are too small (e.g. Profile 4 is only 162 individuals, accounting for 1.32%), which may affect statistical validity (e.g. effect estimates for rare risk categories are not robust). Early factors known to affect lung development, such as birth weight, prematurity, and maternal smoking during pregnancy [2], were not included and may have missed important confounding variables.

The study was observational in design, and it was not possible to determine the causality of risk in childhood in relation to lung function in old age (e.g. reverse causality or unmeasured confounders may have influenced the results) and it based on a single PEF measurement, the trajectory of lung function with age was not tracked, and it was not possible to distinguish between the independent effects of natural aging and risk factors.

This study did not correct for multiple tests of statistical significance (e.g. Bonferroni or FDR) when analyzing associations of multiple risk characteristics with outcomes, increasing the risk of false positives, and reported only subgroup analyses of race and smoking status without delving into other potential effect modifiers (e.g. gender, socioeconomic status).

Despite the association found in this study, the authors directly suggested that ‘early intervention prevents COPD’, without considering the limitations of observational studies that do not validate the effects of interventions [3].

In the discussion section, the clinical significance (e.g. relationship to symptoms or mortality) of PEF <80% as a criterion for impaired lung function in an elderly population is not adequately discussed.

This study revealed the association between respiratory risk profiles in childhood and lung function in old age, innovatively integrating multifactorial analyses, but with limitations in data quality, methodological rigor and extrapolation of findings. Future studies need to incorporate prospective design, multidimensional lung function assessment, and causal inference methods (e.g. Mendelian randomization) to further validate its findings.

## Data Availability

Data sharing is not applicable to this article as no new data were created or analyzed in this study.
